# Combined Analysis of BSA-Seq Based Mapping, RNA-Seq, and Metabolomic Unraveled Candidate Genes Associated with Panicle Grain Number in Rice (*Oryza sativa* L.)

**DOI:** 10.3390/biom12070918

**Published:** 2022-06-29

**Authors:** Yafei Ma, Enerand Mackon, Guibeline Charlie Jeazet Dongho Epse Mackon, Yitong Zhao, Qiufeng Li, Xianggui Dai, Yuhang Yao, Xiuzhong Xia, Baoxuan Nong, Piqing Liu

**Affiliations:** 1 State Key Laboratory of Conservation and Utilization of Subtropical Agro-Bioresources, College of Agriculture, Guangxi University, Nanning 530005, China; mayafei@st.gxu.edu.cn (Y.M.); msmackon@st.gxu.edu.cn (G.C.J.D.E.M.); 1917301052@st.gxu.edu.cn (Y.Z.); 1717303003@st.gxu.edu.cn (Q.L.); 2017301002@st.gxu.edu.cn (X.D.); yuhangyao@st.gxu.edu.cn (Y.Y.); 2 State Key Laboratory of Conservation and Utilization of Subtropical Agro-Bioresources, College of Life Science and Technology, Guangxi University, Nanning 530005, China; breedermackon@st.gxu.edu.cn; 3 Key Laboratory of Rice Genetics and Breeding, Rice Research Institute, Guangxi Academy of Agricultural Science, Nanning 530007, China; xiaxiuzhong@163.com

**Keywords:** rice, grain number, QTL, bulk segregant analysis, transcriptome analysis, metabolism

## Abstract

Rice grain yield is a complex and highly variable quantitative trait consisting of several key components, including the grain weight, the effective panicles per unit area, and the grain number per panicle (GNPP). The GNPP is a significant contributor to grain yield controlled by multiple genes (QTL) and is crucial for improvement. Attempts have been made to find genes for this trait, which has always been a challenging and arduous task through conventional methods. We combined a BSA analysis, RNA profiling, and a metabolome analysis in the present study to identify new candidate genes involved in the GNPP. The F_2_ population from crossing R4233 (high GNPP) and Ce679 (low GNPP) revealed a frequency distribution fitting two segregated genes. Three pools, including low, middle, and high GNPP, were constructed and a BSA analysis revealed six candidate regions spanning 5.38 Mb, containing 739 annotated genes. Further, a conjunctive analysis of BSA-Seq and RNA-Seq showed 31 differentially expressed genes (DEGs) in the candidate intervals. Subsequently, a metabolome analysis showed 1024 metabolites, with 71 significantly enriched, including 44 up and 27 downregulated in Ce679 vs. R4233. A KEGG enrichment analysis of these 31 DEGs and 71 differentially enriched metabolites (DEMs) showed two genes, *Os12g0102100* and *Os01g0580500*, significantly enriched in the metabolic pathways’ biosynthesis of secondary metabolites, cysteine and methionine metabolism, and fatty acid biosynthesis. *Os12g0102100*, which encodes for the alcohol dehydrogenase superfamily and a zinc-containing protein, is a novel gene whose contribution to the GNPP is not yet elucidated. This gene coding for mitochondrial trans-2-enoyl-CoA reductase is involved in the biosynthesis of myristic acid, also known as tetradecanoic acid. The *Os01g0580500* coding for the enzyme 1-aminoclopropane-1-carboxylate oxidase (*OsACO7*) is responsible for the final step of the ethylene biosynthesis pathway through the conversion of 1-aminocyclopropane-1-carboxylic acid (ACC) into ethylene. Unlike *Os12g0102100*, this gene was significantly upregulated in R4233, downregulated in Ce679, and significantly enriched in two of the three metabolite pathways. This result pointed out that these two genes are responsible for the difference in the GNPP in the two cultivars, which has never been identified. Further validation studies may disclose the physiological mechanisms through which they regulate the GNPP in rice.

## 1. Introduction

Rice (*Oryza sativa* L.) is the foremost staple food feeding half of the world’s population and more than half of China’s population [1]. By 2050, the global population will be around 9.7 billion, making the global food and energy demand more challenging than ever. The decrease in suitable agricultural land due to several factors, including climatic changes, leads to strategies to improve the rice grain yields without further expanding farmland and environmental damage [2]. Rice grain yield is a complex and highly variable quantitative trait that depends on three fundamental components: the weight of grain, the number of effective panicles per unit area, and the grain number per panicle (GNPP), each controlled by multiple genes (QTL). The GNPP has been revealed as the primary contributor to total yield per unit area and is essential for its improvement [3].

The physiological network and genes underlying panicle and grain formation have been thoroughly studied. The primary mechanism includes the initiation during which the shoot apical meristem (SAM) is transformed to inflorescence meristem (IM), the formation of rachis branches, and spikelet specialization successively. These steps are vital in the panicle architecture and GNPP [4,5]. Recent studies highlighted several hormones, including cytokinin (CK), gibberellin acid (GA), abscisic acid (ABA), and ethylene (ET), that interfere to regulate the transcriptional and post-transcriptional activities of genes in rice, acting in different pathways involved in these processes [6,7,8,9]. Numbers of genes have been identified from high-throughput QTL mapping, and within the past ten years, the number of the QTLs related to the GNPP doubled from 315 QTL [10], 369 [11] to 722 QTL (http://www.gramene.org; accessed on 10 April 2022) distributed along the 12 chromosomes in the rice genome. The first QTL grain number 1a (*GN1a*) was mapped and cloned on chromosome1 coding for a cytokinin oxidase (*OsCKX2*) which repressed the GNPP by reducing CK content [12], and with aberrant panicle organization1 (*APO1*) controlling the proliferation of cells in the meristem [4]. These works set the foundation for GNPP-related gene mapping. CK is essential in rachis branch formation, and the high expression of *OsCKX2* weakened CK signaling in IM leads to few rachis branches and GNPP [13,14]. Besides regulating GA homeostasis, *OsCYP71D8L* negatively controls the length of the panicles, and the rachis branch numbers, and subsequently the rice GNPP [15]. Major QTL which controls, directly or indirectly, the GNPP and related traits include: heading date 7 (*Ghd7*), which increases the differentiation period [16]; *LAX* regulating the rachis branches initiation [17]; dense erect panicle1 (*DEP1*) controlling the secondary branches of inflorescence and GNPP [18]; grain number 4-1 *(GN4-1*) and grain number per panicle1 (*GNP1*) regulate the number of rachis branches and GNPP [10,19]; LONELY GUY *(LOG*) positively regulates GNPP by controlling the concentration and distribution of CK [13]; and Pyrabactin Resistance-Like *(PYL*) positively regulates ABA signaling and negatively regulates GNPP in rice [7].

The development of new molecular technology allowed the transition from the conventional method, and the integration of omics tools facilitated the identification of new functional genes and pathways. During the last decade, most of the studies for mapping QTL relied on traditional methods, which involve the construction of mapping the population through the F_2_ generation, recombinant inbred lines (RIL), and near-isogenic lines (NIL), followed by the drawing of a genetic linkage map, and cloning. The following is a strategy by which numerous genes have been mapped and cloned. The most relevant include *qGN1c* mapped using a set of chromosomal segment substitution lines (CSSLs) [20], *SPP1* identified through NILs controlling the number of spikelets [21], *qTGW3.2* for grain weight in rice using RIL [22], *GNP1* [19], and *qgnp7*(*t*) [23] governing GNPP revealed through map-based cloning, and two steps substitution mapping, respectively. However, traditional gene mapping is tedious and time-consuming; most QTLs are inconsistent and span large genomic regions, making identifying candidate genes for a trait more challenging. New strategies which combine traditional methods and emerging technologies such as high-throughput whole-genome sequencing (NGS), alternative sequencing analysis, mapping by sequencing, bulked segregants analysis (BSA), RNA-sequencing (RNA-seq), and genome-wide association study (GWAS), have accelerated the identification of candidate genes for complex characters, and several studies have been successfully conducted to identify a new gene [2,24,25,26,27]. BSA-Seq and RNA profiling were coupled to reveal the pathway and genes associated with the heading type in Chinese cabbage [28], chilling tolerance in rice [24], and plant architecture in *Brassica napus* [29]. Likewise, significant gene candidates have been identified by combining sequencing and a metabolome analysis in albino jackfruit seedlings [30] and anthocyanin in cucumber fruit skin [31].

Although comprehensive studies on QTL mapping for the GNPP have been carried out, discovering new QTL remains an excellent value for molecular and practical breeding. Considering the complexity of grain number related-genes and the limitation of traditional QTL mapping technology, in this study, we successfully integrated the BSA-seq, RNA-Seq, and metabolome analysis to identify two new genes linked to the GNPP.

## 2. Materials and Methods

### 2.1. Plant Materials and Growth Conditions

**Figure 1:**
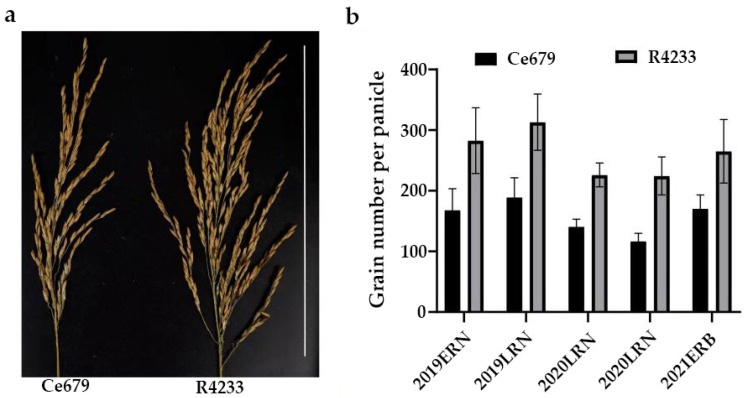
Analysis of the panicle structure between R4233 and Ce679. (**a**) Main panicle structure of Ce679 and R4233. The white line on the right side of the figure represents the scale bar in 25 cm length. (**b**) Histogram of the grain number per panicle in Ce679 and R4233. Data were collected over three years at two different sites. The site of Nanning (22°48′ N, 108°22′ E) during the years 2019–2020, and Bobai (22.27° N, 109.98° E) during the year 2021. For each trial, ten plants were harvested from Ce679 and R4233, the total grain number was counted, and the number of panicles per plant was evaluated from an individual plant. The grain per panicle was obtained by dividing the total grain per plant by panicles per plant. The data presented here are the means with SD (*n* = 10). ERN and LRN denote early rice and late rice in Nanning, respectively; ERB denotes early rice in Bobai.

In this work, two *indica* rice varieties from Guangxi province, China, were used, including Ce679 with a low grain number and R4233 with a high grain number. Ce679 is a restorer line developed from a common wild rice and IR661 and IR2061 (IR661∥IR2061/Hepu wild rice). It has strong lodging resistance, high combining ability, good rice quality, and the strong cold resistance of Hepu wild rice. R4233 is a restorer line developed through three generations of backcrossing of Ce679 and F_1_ (Ce679∥75-1-172/C4064). After several tests, the R4233 restorer line contained the blast resistance gene *Pi9* (from 75-1-172) and a high grain number compared to all other parents. Crossing between Ce679 and R4233 generated 436 F_2_ segregating populations from which the genomic regions associated with the GNPP were identified. The parents Ce679 and R423 were grown in two different sites to evaluate if the environment will affect the GNPP, and assays were conducted from 2019 to 2021 during the two yearly planting seasons known as early season (15 March–15 July) and late season (20 August to 20 November). Phenotypic evaluations of grain number per panicle in the F_2_ population were carried out during the period 2019–2020 (four seasons: two early and two late seasons) in an experimental field at Guangxi University, Nanning city, Guangxi province, China (22°48′ N, 108°22′ E), and the early season from March to August 2021 in Bobai, Guangxi province (22.27° N, 109.98° E), respectively (Figure 1) with different climates. The experimental design was a randomized complete design. In total, three plots (two for the two parents and one for the F_2_) of 1 m × 12 m and 1 × 18 m were designed. The distance between plants was 20 × 20 cm and each plot had 300 plants for parents and 436 plants for F_2_.

### 2.2. Methods

#### 2.2.1. Phenotypic Evaluation

To investigate agronomic traits, we grew the two varieties in the field. Ten individuals were chosen from each parent after maturation. Different traits include panicle length, filled grain number, total grain number, number of tillers, secondary and primary branches, GNPP, grain length, thickness and width, and thousand kernel weight. After the crossing, the number of panicles and grain per plant were evaluated in the F_2_ population. We obtained the GNPP by dividing the total grain number of a plant by the total panicle number. Statistical analyses were performed using a *t*-test. To analyze the variance and compare the mean differences (*p* ≤ 0.05), we used SigmaPlot software v. 125 (Systat Software Inc., San Jose, CA, USA) and Duncan’s multiple range test, respectively.

#### 2.2.2. Sample Collection, Extraction of the Genomic DNA, and Construction of Segregating Pools

For the BSA-seq analysis, leaves were collected in April 2020 for all 436 F_2_ plants, labelled, and kept in −80 °C. After maturation, the grain number from different plants was recorded, the frequency distribution was drawn, and samples were chosen for DNA extraction. The samples for the RNA-seq and metabolome analysis were collected in May 2021 from young panicles, uniform in length (≤2 cm) [32], and disease-free during the harvesting period. Upon collection, the young panicles were snap-frozen, then stored in the freezer at −80 °C for further experiments. The experimental design was made of three biological replicates to reduce errors.

DNA extraction was performed through the cetyltrimethylammonium bromide (CTAB). Briefly, 0.5 g of leaf sample was collected from each offspring F_2_ individual deriving from Ce679 and R423 crossing, mixed, ground in liquid nitrogen into a powder form in the 2 mL centrifuge tube using a grinder. Then, 800 μL 2% preheated CTAB extraction buffer (CTAB-4 g; NaCl-16.34 g; 1 M Tris-HCl-20 mL (PH 8.0); 0.5 M EDTA-8 mL; PVP-360-2 g volume to 200 mL (pH 8) re-sterilization, preheated in a water bath to 65 °C for 30 min was added and incubated in Mary’s bath at 65 °C for 40 min with intermittent shaking every 10 min. Then, one volume (400 μL) of chloroform-isoamyl alcohol (24:1) was added, thoroughly mixed by inverting the tube for 5 min before centrifugation (12,000× *g*, 5 min). The suspension was aspirated carefully and transferred to a new 1.5-mL centrifuge tube. Afterward, twice the volume of isopropyl-alcohol was added, mixed gently, and stood at −20 °C for over 30 min. The nucleic acid in the aqueous phase was pelleted after centrifugation (12,000× *g*, 5 min). A 0.5 mL volume of 70% ethanol was added to the precipitate, and centrifuged (12,000× *g*, 5 min) after 5 min at room temperature. The above wash was repeated. The sample was air-dried and dissolved in 50 μL sterilized deionized water. DNA concentration was estimated by the Nanodrop 1000 spectrophotometer and run on 1% agarose gel electrophoresis to assess purity. DNA from 30 plants representing high, middle, and low grain numbers were equally mixed to form H, M, and L pools.

#### 2.2.3. Bulk Segregants Analysis Sequencing

We prepared five DNA libraries from the two parents and three constructed pools, followed by sequencing. Briefly, DNA was fragmented by ultrasonication into small pieces of 350 bp, ligation with the adapters, and then purified. Further, the Illumina HiseqTM2000 platform (Beijing Biomarker Biotechnology Co., Beijing, China) was used to sequence the DNA. We filtered data to obtain high-quality reads to ensure successful progress in the subsequent analysis. Data filtering involved trimming the base with low quality, removing the reads with 50% bases with a Q-score less than 10, and those with more than 10% missing bases. The clean reads obtained after filtering were mapped to the reference genome (*Oryza_sativa*_IRGSP-1.0) using BWA software [33]. Subsequently, we performed SNP calling and annotation using GATK tools and SnpEff software [34,35]. Reads were removed on the reference genome with the Mark Duplicates tool in Picard (http://sourceforge.net/projects/picard/; accessed on 28 October 2020). The local rearrangement and base mass value calibration was carried out to detect SNP and small indels (1–5 bp) using the GATK software package [36].

Further, we used SnpEff software to perform SNP annotation and determine the impacts (synonymous and non-synonymous mutations) of small indels in the genome [35]. The candidate regions of the genome associated with the GNPP were identified, and the differences in allele frequency between bulked pools were performed with the SNP-index algorithm [34,37]. The SNP index was called the scale of short reads containing SNPs different from the reference genome [38]. The Δ(SNP-index) was referred to as the SNP-index difference between H-pool and L-pool, H-pool and M-pool, and M-pool and L-pool and was used to calculate the 1000 permutations in the genome with 95% confidence within the candidate regions of the GNPP [2]. The distribution of the SNP index among the genome within 1 Mb width windows and 1 kb at each step was calculated through the sliding window analysis. The above analysis was carried out through the online platform BMKCloud (http://www.biocloud.com/; accessed on 4 November 2020).

#### 2.2.4. RNA-Sequencing and Gene Profiles Analysis

Fresh young panicles about 2 cm [32] were collected from different plants, and 0.5 g were used for total RNA isolated using a TRIzol^®^ reagent kit (Invitrogen, Carlsbad, CA, USA) according to the manufacturer’s protocol. RNA concentration, purity, and integrity were evaluated using an Agilent 2100 Bioanalyzer (Agilent Technologies, Palo Alto, CA, USA) and agarose gel electrophoresis. We further generated a cDNA library following the Invitrogen protocol. The polyA selection method by oligo (dT)-attached magnetic beads was used to isolate and enrich mRNA from total RNA.

Afterward, the enriched mRNA was fragmented into small pieces of 350 bp. This experiment was performed by adding a fragmentation buffer. The fragments were reverse transcribed into the first-strand cDNA with random primers. The second-stranded cDNA was synthesized by reverse transcriptase and purified with a QiaQuick PCR extraction kit (Qiagen, Venlo, The Netherlands). Subsequently, the cDNA structure was end-repaired, a poly (A) tail was added, and the index adaptor was ligated to prepare hybridization. The ligated products were selected based on the size after running on 2% low-range ultra-gel electrophoresis (Certified Low Range Agarose, Bio-Rad, Shanghai, China). The PCR amplification was performed for 15 cycles and sequenced using Illumina Hiseq2500 by Gene DeNovo Biotechnology Co., Guangzhou, China.

Quality control of raw data was carried out before data analysis. We generated raw reads in the FASTQ format. Then, low-quality reads and adapters were filtered to obtain the clean reads. Subsequently, we used HISAT2 2.4 with RNA-strandedness and the default parameter [39] to assemble and map high-quality reads to the reference genome (http://plants.ensembl.org/Oryza_sativa_IRGSP-1.0; accessed on 9 May 2021). Fragments per kilo-base of transcript per million (FPKM) were estimated to quantify the gene expression levels [39]. The differentially expressed genes (DEGs) were analyzed through DESEeq2 software [40]. Multiple hypotheses with the *p*-value thresholds of fold change (FC) ≥ 2 and false discovery rate (FDR) ≤ 0.05 were applied. Gene ontology was performed with the GOSeq R package. Gene function and utilities of the biological system were annotated based on the Kyoto Encyclopedia of Genes and Genomes (KEGG; http://www.genome.jp/kegg; accessed on 20 May 2021) [41]. The KEGG enrichment analysis of DEGs has been performed using KOBAS software [42]. All the analyses above were performed using free online data analysis OmicShare tools (https://www.omicshare.com/tools; accessed on 25 May 2021).

#### 2.2.5. Metabolomes Analysis

##### Sample Preparation and Extraction of Metabolites

The samples were freeze-dried and crushed using a mixer mill (MM 400, Retsch, Haan, Germany) with a zirconia bead for 1.5 min at 30 Hz. In total, 90–105 mg of powder was weighed and extracted overnight at 4 °C with 1 mL of 70% aqueous methanol containing 0.1 mg/L lidocaine for the internal standard. Samples were then centrifuged at 10.000 g for 10 min. The supernatant was absorbed and filtered (SCAA-104, 0.22 μm pore size; ANPEL, Shangai, China, www.anpel.com.cn/; accessed on 25 May 2021) before the liquid chromatography-mass spectrophotometer (LC-MS/MS) analysis. All samples’ quality controls (QC) were performed to detect the experiment’s reproducibility and ensure that a scientific process met the qualitative and quantitative criteria. The QC samples were analyzed intermittently for the duration of the analytical study to assess the variance observed in the data throughout the sample preparation, data acquisition, and pre-processing steps. Replicate injections should provide comparable data for each injection; however, an analytical variance was be observed, and the replicate QC injections were used to measure this variance across the analytical study.

##### AB Sciex QTRAP4500 (UPLC) Analysis

The compounds extracted were analyzed using an LC-ESI-MS/MS system (UPLC, Shim-pack UFLC SHIMADZU CBM30A, http://www.shimadzu.com.cn/; accessed on 30 May 2021; MS/MS (Applied Biosystems 6500 QTRAP, http://www.appliedbiosystems.com.cn/; accessed on 30 May 2021) [43]. For this assay, 2 μL of samples was injected onto a waters ACQUITY UPLC HSS T3 C18 column (2.1 mm × 100 mm, 1.8 µm) operating at 40 °C and a flow rate of 0.4 mL/min. Two mobile phases were employed: phase A, composed of acidified water (0.04% acetic acid); and phase B, composed of acidified acetonitrile (0.04% acetic acid). Then, compounds were separated using the following gradient: 95:5 phase A/phase B at 0 min; 5:95 phase A/phase B at 11.0 min; 5:95 phase A/phase B at 12.0 min; 95:5 phase A/phase B at 12.1 min; 95:5 phase A/phase B at 15.0 min. The effluent was connected to an ESI-triple quadrupole ion trap (Q TRAP)-MS.

LIT and triple quadrupole (QQQ) scans were acquired on a triple quadrupole-linear ion trap mass spectrometry (Q TRAP), AB Sciex QTRAP6500 system, equipped with an ESI-Turbo Ion-Spray interface, operating in a positive ion mode and controlled by Analyst 1.6.1 software (AB Sciex). The operation parameters were as follows: ESI source temperature 500 °C; ion spray voltage (IS) 5500 V; curtain gas (CUR) 25 psi; the collision-activated dissociation (CAD) was set the highest. QQQ scans were acquired as MRM experiments with optimized declustering potential (DP) and collision energy (CE) for each MRM transition. The *m*/*z* range was set between 50 and 1000.

##### Data Processing, Annotation, and Metabolites Identification

The data filtering, peak detection, alignment, and calculations were performed using Analyst 1.6.1 software. Metabolites were identified by searching an internal database and public databases (MassBank, KNApSAcK, HMDB [44], MoTo DB, and METLIN [45]). Then the *m*/*z* values, the RT, and the fragmentation patterns were compared with the standards. Gene Denovo Biotechnology Co., Ltd. (Guangzhou, China) conducted the processing and annotation of our metabolomic data. In the preliminary visualization of differences between different groups of samples, we applied the unsupervised dimensionality reduction method principal component analysis (PCA) in samples using R package models for the multivariate analysis (http://www.r-project.org/; accessed on 4 June 2021). For an analysis of differential metabolite profiles, we applied variable importance in the projection (VIP) score with 1 set as the threshold of the (O)PLS model, which allowed us to rank the metabolites that best distinguished between two groups. Differential metabolites were screened between two groups using a *t*-test as a univariate analysis, and those with *p* ≤ 0.05, VIP ≥ 1 were considered differentially expressed. Metabolites were mapped to KEGG metabolic pathways (http://www.kegg.jp/kegg/pathway.html; accessed on 9 June 2021) to identify the pathway enrichment and were annotated using the KEGG compound database (https://www.kegg.jp/kegg/compound/; accessed on 30 June 2021). The pathway enrichment analysis identified significantly enriched metabolic pathways or signal transduction pathways in differential metabolites compared with the whole background. The calculated *p*-value was generated through an FDR correction, taking FDR ≤ 0.05 as a threshold. Pathways fitting this condition were defined as significantly enriched pathways in differential metabolites.

#### 2.2.6. Expression Analysis of Putative Grain Number Genes by Reverse Transcription Quantitative PCR (RT-qPCR)

To evaluate the gene expression, we performed an RT-qPCR. The total RNA was isolated from rice young panicle using the EASYspin RNA Rapid Plant kit (RA106-02, Biomed, www.biomed168.com; accessed on 10 June 2022) according to the manufacturer’s instructions. We chose six candidate genes differentially expressed in Ce679 vs. R4233 including *Os01g0600900*, *Os12g0102100*, *Os01g0580300*, *Os01g0580500*, *Os01g0591000*, and *Os01g0589000*. Rice OsActin1 was used as an internal reference gene to normalize the gene expression level. The primer sequences listed in Table S1 were retrieved from https://biodb.swu.edu.cn/qprimerdb/best-primers-ss; accessed on 10 June 2022. First-strand full-length cDNAs were synthesized from 2 µg of total RNA using the StarScript II First-strand cDNA Synthesis Mix with gDNA Remover (A224-05, GenStar, Beijing, www.gene-star.com; accessed on 10 June 2022) according to the manufacturer’s instruction. The RT-qPCR was carried out using the ChamQ Universal SYBR qPCR Master Mix (Q711-02, Vazyme, China, www.vazyme.com; accessed on 12 June 2022) on the QTOWER3G (Germany) according to the manufacturer’s instructions. Six biological repeats were used for the treatment (R4233) and control (Ce679) for gene expression profiles. The reaction was adjusted following the thermal cycling conditions as the initial denaturing temperature, 95 °C for 1 min, followed by 40 cycles, and each cycle consisted of 95 °C for 5 s and 60 °C for 30 s. The gene expression level was calculated by the 2^−ΔΔCt^ calculation method.

## 3. Results

### 3.1. Phenotypic Evaluation of R4233 and Ce679

In all trials in Nanning and Bobai, R4233 demonstrated a high grain number compared to Ce679 and the mean value of the GNPP over the three years was 262.58 and 158.29, respectively. The GNPP in R4233 was nearly 1.66-fold more than that in Ce679. Other important agronomic traits were also investigated in the Nanning site from August to December 2020, as illustrated in Table 1. Our results showed a significant difference (*p* ≤ 0.01) in flag leaf length, number of secondary branches per the main panicle, grain length, grain number per panicle, length–width ratio, and grain yield per plant R4233 and Ce679. In contrast, no significant differences in panicle length, plant height, tiller number, seed setting ratio, and grain width were identified (Table 1).

We evaluated the agronomic parameters in hybrid F1 from the crossing between Ce679 and R4233. The results showed a significant difference in the number of secondary branches per panicle, and also the grain number per panicle at 5% when we compared Ce679 and F1. A similar result was also obtained between R4233 and F1. However, it was higher in F1 and R4233 compared to Ce679 (Table 2). We highlighted that the number of secondary branches per panicle was significantly higher (*p* ≤ 0.05) than the primary branches in both parents and F1. This result suggested that the secondary branches were the highest contributor to the total branches per panicle. We found that in Ce679, the primary and secondary branches per panicle contributed 17.7 and 82.3% to the total branches per panicle. In R4233 and F1, this contribution was 13.4 and 86.6% and 12.9 and 87.1%, respectively.

### 3.2. Variation of the GNPP in F_2_ Population and the Construction of the Segregating Pools

**Figure 2:**
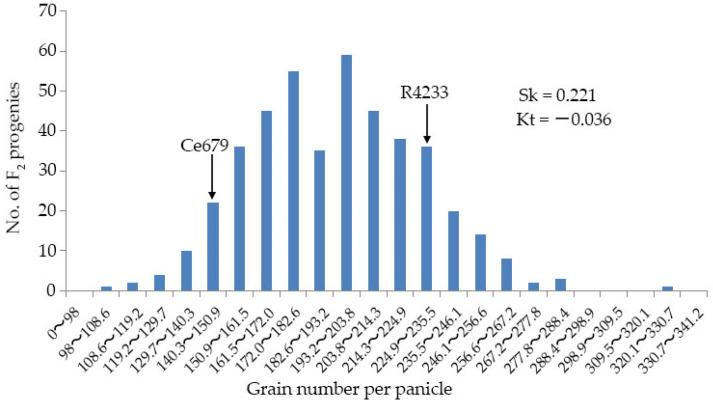
Frequency distribution of GNPP of the progenies derived from the crossing between Ce679 and R4233. The back arrows show the mean values of the distribution in Ce679 and R4233.

**Figure 3:**
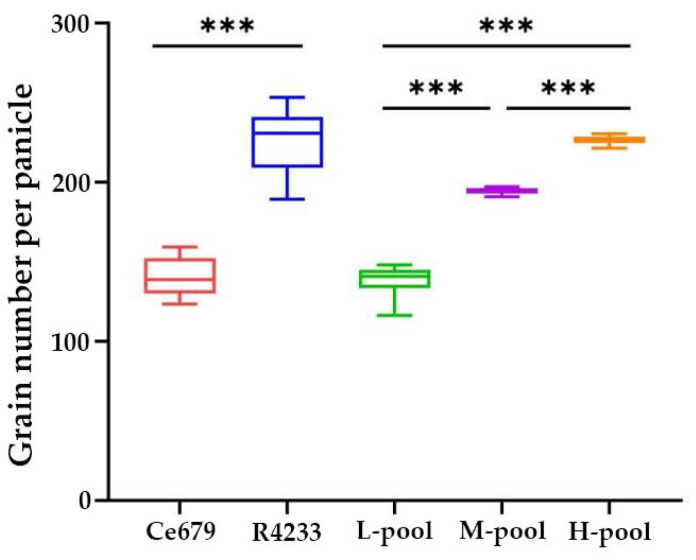
Box plots representing GNPP of the three BSA pools and parents. L-pool, M-pool, and H-pool show low, middle, high GNPP extremities, respectively, with the median indicated by the band inside the box. *** indicates *p* ≤ 0.001.

The GNPP of individual plants in the F_2_ ranged between 98.11 and 320.20. In Ce679 and R4233, it was found to be 140.90 and 226.05, respectively. As shown in Figure 2, the GNPP was dispersed with two peaks surrounded by the two mean values, indicating that the GNPP as a quantitative trait was controlled by multiple genes (QTL). In total, 30 individuals with the high grain number (GNPP: 221.5~230.43), 30 individuals with the middle grain number (GNPP: 190.8~197.17), and 30 individuals with a low grain number (GNPP: 116.33~148) were used to build the H-pool, M-pool, and L-pool, respectively (Table S2). The average of the GNPP was highest in the H-pool (226.16), followed by the R4233 (226.05), M-pool (194.41), and Ce679 (140.89); it was lowest in the L-pool (138.85) (Figure 3).

### 3.3. Analysis of BSA-Seq Data and Reads Assembly

We constructed five cDNA libraries for BSA-seq and conducted the Illumina HiSeq platform. Through RNA-seq, 263.7 million (M) raw reads were generated. After data filtering, 41.35~71.35 M clean reads (>98%) were obtained from a different sample. Further, the clean reads were mapped to the reference genome, and the proportion of mapped reads to clean reads was 97.85%, 97.57%, 97.48%, 97.77%, and 97.12% in Ce679, R4233, L-pool, M-pool, and H-pool, respectively, with the sequencing depth ranging between 30 and 49 folds. The results showed that the sequencing depth was relatively close to each other in segregating pools and the parents, consistent with the accuracy of the BSA analysis. The one-fold coverage ratio ranged from 91.43 to 94.24% (Table 3). Furthermore, SNPs and indels, including homozygotes and heterozygotes, were investigated. Our analysis showed 710,891, 176,765, 120,929, and 182,133 SNPs, including 18,265, 3075, 1797, and 3269 non-synonymous SNPs in Ce679 vs. R4233, L-pool vs. H-pool, L-pool vs. M-pool, and M-pool vs. H-pool, respectively. Meanwhile, we identified 173,695, 48,473, 36,704, and 51,080 indels in Ce679 vs. R4233, L-pool vs. H-pool, L-pool vs. M-pool, and M-pool vs. H-pool, respectively. These indels were divided into 1919, 717, 591, and 681 frame-shift indels in the Ce679 vs. R4233, L-pool vs. H-pool, L-pool vs. M-pool, and M-pool vs. H-pool, respectively (Tables S3 and S4). The SNPs and indels’ densities were evaluated in different chromosomes and the result was similar among the three pools, which varied from 0.00559 to 0.00741 per bp and 0.00127 to 0.00165 per bp. In all pools, the highest SNP and indel density was observed in chromosome 12 and 11, while the lowest was in chromosome 4 (Tables S5 and S6). High-density single nucleotide polymorphisms (SNPs) were used as highly favored makers to analyze genetic diversity and population structure, to construct high-density genetic maps, and to provide genotypes for genome-wide association analysis.

### 3.4. Identification of the Candidate Regions Related to the GNPP

**Figure 4:**
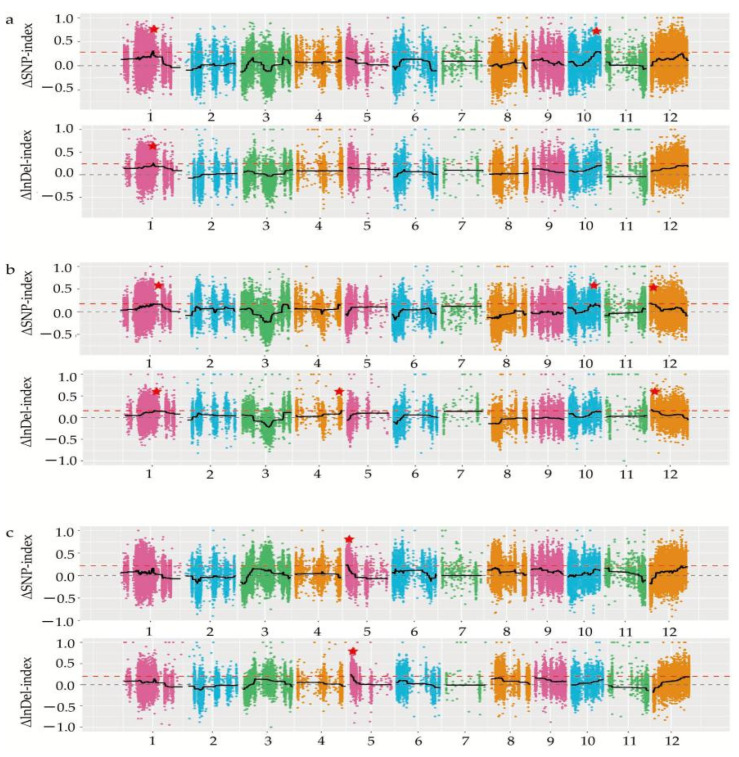
Manhattan plot on the variation tendency of the Δindel-index and ΔSNP-index among L-pool, M-pool, and H-pool associated with GNPP distribution in different chromosomes. (**a**), Δindel-index and ΔSNP-index between L-pool and H-pool; (**b**), Δindel-index and ΔSNP-index between L-pool and M-pool; (**c**), Δindel-index and ΔSNP-index between M-pool and H-pool. Red stars indicate the candidate regions associated with the GNPP. The number on *x*-axis represents the chromosome number. The values of Δindel-index or ΔSNP-index were calculated and plotted with colored dots. The fitted Δindel-index or ΔSNP-index is shown with the black line, and the threshold line for the 99th percentile is indicated with a red line.

The INDEL and SNP index, which represent the population’s parental allele frequency, were used to calculate the candidate regions of the genome related to the GNPP. The Δindel and ΔSNP-index were associated with the genomic position. As illustrated in the Manhattan plots (Figure 4), the peak regions above the red lines (99%, threshold value) represent the regions where the GNPP may be associated. The ΔSNP-index method identified three, eight, and one candidate regions associated with the GNPP on chromosomes 1 and 10, 1, 10, and 12, and 5, in L-pool vs. M-pool, and M-pool vs. H-pool. Its total size was 3.72, 1.06, and 1.21 Mb, and it included 514, 180, and 178 annotated genes in L-pool vs. H-pool, L-pool vs. M-pool, and M-pool vs. H-pool, respectively (Table S7). According to the ΔIndel-index method, three candidate regions in the genome were distributed on chromosome 1, 12, and 5, respectively, with a total size of 0.41, 0.31, and 0.94 Mb and contained 38, 44, and 143 annotated genes in L-pool vs. H-pool, L-pool vs. M-pool, and M-pool vs. H-pool, respectively (Table S8). The candidate regions identified from these two methods were intersected, and the final association regions were determined. This region spanned 5.38 Mb and contained 739 annotated genes (Table S9). As depicted in Table 4, three associated regions were identified on chromosome 1, covering 0.41 Mb (22.29~22.70 Mb), 0.06 Mb (22.83~22.89 Mb), and 0.75 Mb (22.93~23.68 Mb), and included 38, 6, and 81 genes, respectively. The other three regions were distributed on chromosomes 10, 12, and 5, which had a size of 2.91 Mb, 0.31 Mb, and 0.94 Mb, and it included 427, 44, and 143 genes, respectively (Table 4).

### 3.5. Gene Expression Profile Analysis and Identification of Candidate Genes in the Final Associations’ Regions

**Figure 5:**
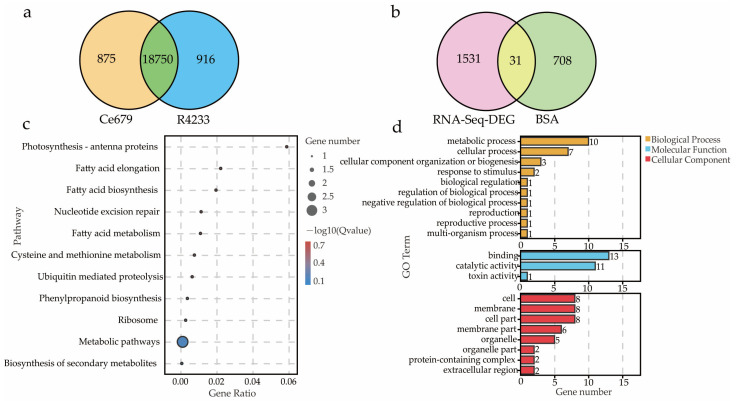
Gene expression pattern and DEGs analysis for GNPP in candidate intervals. (**a**), Venn diagram of expressed genes; (**b**), Venn diagram of BSA-Seq merged with RNA-seq; (**c**), KEGG pathway enrichment analysis; (**d**), GO classification.

The RNA-sequencing of the six cDNA libraries was generated after filtering a total of 78.77 Mb clean reads with the average GC content of nearly 49.08%; Q20 and Q30 were 97.95% and 94.26%, respectively (Table 5). Further, using HISAT2 software, 93.95 to 94.62% of the clean reads were mapped to the reference genome *Oryza sativa* L. ssp. Japonica. Our analysis detected 19,625 and 19,666 genes in Ce679 and R4233, respectively, in which 18,750 genes were commonly expressed in Ce679 and R4233 (Figure 5a). A subsequent analysis showed 1562 differentially expressed genes (DEGs) between Ce679 vs. R4233, with 824 upregulated and 738 downregulated (Figure 5b).

The BSA-Seq and RNA-Seq results suggested that among all the genes identified, 738 genes were expressed in the young panicle (Table S10), of which 31 genes (more than 4% of the 738 genes) were differentially expressed (Table S11). We investigated GO-enrichment to predict the biological function of different DEG sets and revealed that 31 DEGs belonged to three categories: biological process, cellular component, and molecular function. Most of the DEGs were assigned to metabolic, cellular, and cellular component organization or biogenesis processes of the biological process category (Figure 5d). In addition, it was found that the cell, cell parts, and membrane in the cellular component and binding, catalytic, and toxin activity were enriched in the molecular function category. These 31 DEGs were used for the KEGG analysis, and the results indicated that 11 pathways were significantly enriched (Figure 5c). These pathways contained six DEGs (*Os01g0580300*, *Os01g0580500*, *Os01g0589000*, *Os01g0591000*, *Os01g0600900*, and *Os12g0102100*) (Table 6). *Os01g0580500*, known as *OsACO7*, aminocyclopropane-1-carboxylate oxidase gene, was related to Ethylene biosynthesis.

### 3.6. Metabolites Associated with the Young Panicle of Rice

**Figure 6:**
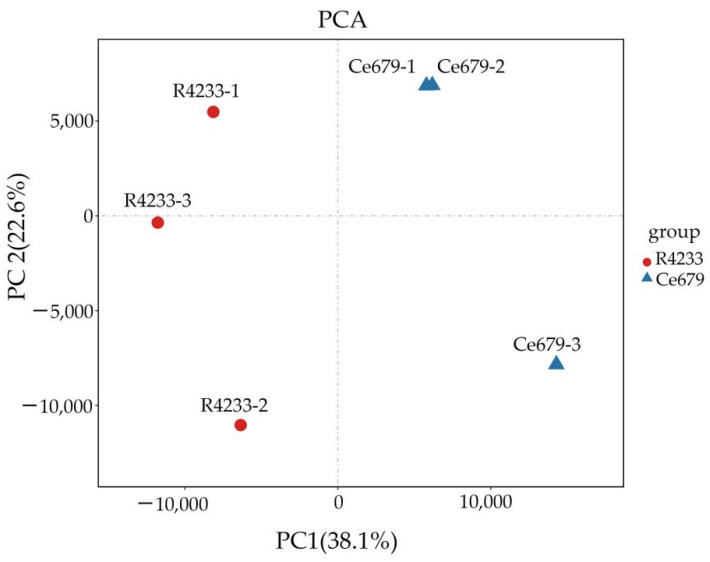
PCA of detected peaks in a young panicle of Ce679 compared with R4233 through LC-MS/MS. PCA scores were derived from the metabolites detected in the three replicated samples.

The principal component analysis (PCA) approach allowed the comparison of metabolite peaks detected through the LC-MS/MS method in the young panicle of Ce679 and R4233. The samples were separated according to PC1 (38.1%) and PC2 (22.6%) (Figure 6). The PCA plot showed a separation between the two varieties, suggesting a discrepancy in their metabolites in line with the phenotypic difference.

### 3.7. Combined Analysis of the Differential Accumulated Metabolites (DAMs) and DEGs

**Figure 7:**
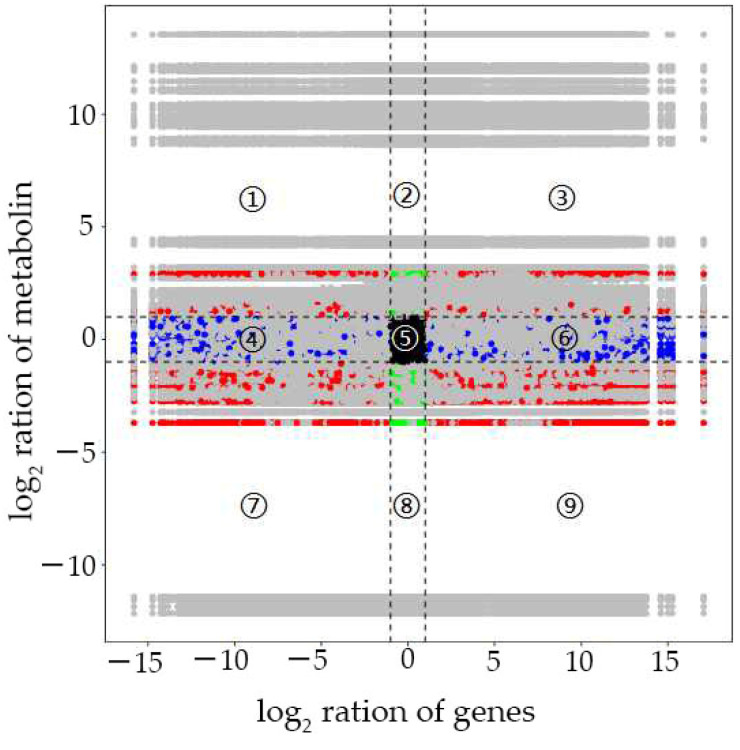
Quadrant diagrams representing the association of the DAMs and DEGs between Ce679 and R4233. The *x*-axis indicates the log_2_ ratio of genes, and the *y*-axis the log_2_ ratio of metabolites. Black dotted lines indicate the threshold. Each gene indicates a metabolite or gene. Black dots denote the unchanged genes or metabolites; green dots indicate DAMs with unchanged genes; blue dots represent DEGs with unchanged metabolites; DEGs and DAMs are shown by red dots, divided into 9 quadrants from top to bottom and left to right with black dotted lines. The quadrants ①, ②, and ④ indicate that the expression abundance of metabolites is higher than that of genes; the quadrants ③ and ⑦ indicate that the expression patterns of genes are consistent with the metabolites; the quadrant ⑤ indicates that the genes and metabolites are not differentially expressed; the quadrants ⑥, ⑧, and ⑨ denote that the expression abundance of metabolites is lower than that of genes.

We performed a correlation analysis on DAMs and DEGs. Nine quadrant diagrams were drawn, elucidating the metabolites’ variations and their corresponding genes with a Pearson correlation coefficient over 0.99, and the correlation coefficient clustered heat map (Figure 7). It showed that quadrants 9 and 7 had more DAMs and DEGs than other quadrants.

The correlation analysis was carried out on the differentially accumulated metabolites (DAMs) and DEGs. The variations in the metabolites and their corresponding genes with the Pearson correlation coefficient over 0.99 were selected to draw nine quadrant diagrams and the correlation coefficient clustered heat map. As shown in Figure 7, the higher number of DAMs and DEGs were in the seventh and ninth quadrants. A positive correlation between DAMs and DEGs were observed in quadrant 9, while a negative was observed in quadrant 7.

**Figure 8:**
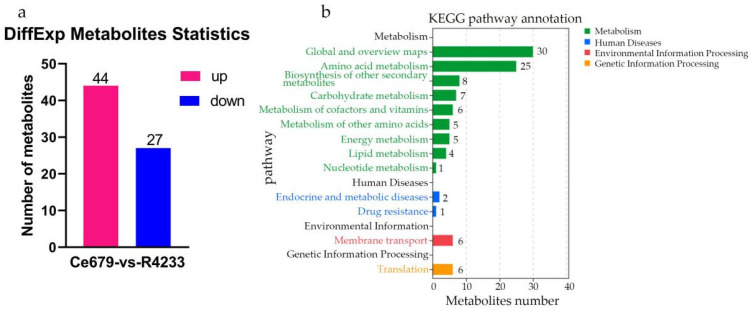
Identification of DEM and KEGG analysis. (**a**), DEMs of young panicle in Ce679 and R4233; (**b**), KEGG annotation of DEMs from young panicle in Ce679 and R4233.

By comparing replicated samples from Ce679 and R4233, the differentially expressed metabolites (DEMs) were highlighted. Globally, 1024 metabolites were identified, including 27 downregulated and 44 upregulated in Ce679 vs. R4233 (Figure 8a). The levels of pme0008, mws0473, mwsmce257, pmb3042, Lmmn002260, mws1346, and Hmpn005101 were significantly different in the two varieties (Table S12). We found that the level of Hmpn005101 was more than three times higher in Ce679 than in R4233, and Lmmn002260 contents were twice higher in Ce679 than in R4233. Subsequently, the KEGG annotation revealed that the categories “global and overviewed maps”, “amino acid metabolism”, and “biosynthesis of other secondary metabolites” were the more represented pathways (Figure 8b).

**Figure 9:**
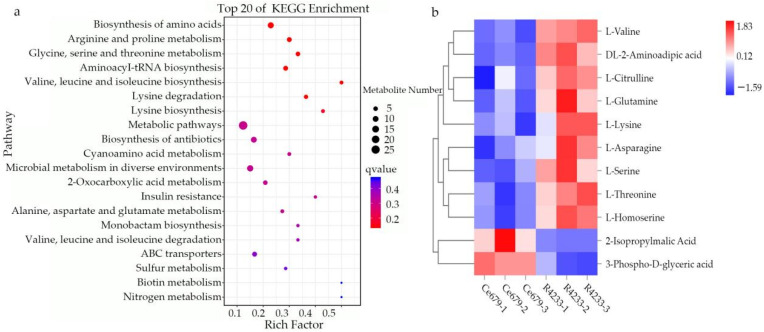
KEGG pathway of DEM and hierarchical clustering analysis. (**a**), KEGG analysis of DEMs; (**b**) hierarchical clustering analysis of DEMs associated with biosynthesis of amino acids (Padj ≤ 0.05, and log_2_ FC > 1) in a young panicle.

The DEM was significantly enriched in biosynthesis-related KEGG pathways, including the biosynthesis of amino acids, arginine, and proline metabolism (*p* ≤ 0.05; Figure 9). The biosynthesis of amino acids was the most significantly enriched pathway. The L-Serine, L-Valine, L-Threonine, L-Homoserine, L-Asparagine, L-Glutamine, L-Lysine, DL-2-Aminoadipic acid, L-Citrulline, 2-Isopropylmalic Acid, and 3-Phospho-D-glyceric acid metabolites were found to be related to the biosynthesis of amino acids; however, γ-Aminobutyric acid, 4-Guanidinobutanal, N-Acetylputrescine, Agmatine, 4-Acetamidobutyric acid, and 4-Guanidinobutyric acid were related to the arginine and proline metabolism (Figure 9).

### 3.8. Genes Associated with the GNPP in a Young Panicle

RNA-Seq data from young panicles were mapped to the reference genome *Oryza Sativa* Japonica (http://ftp.ensemblgenomes.org/pub/plants/release-49/fasta/oryza_sativa/dna/, accessed on 9 March 2021); 94.61% and 94.54% of reads were successfully mapped from R4233 and Ce679 samples, respectively (Table 7).

**Figure 10:**
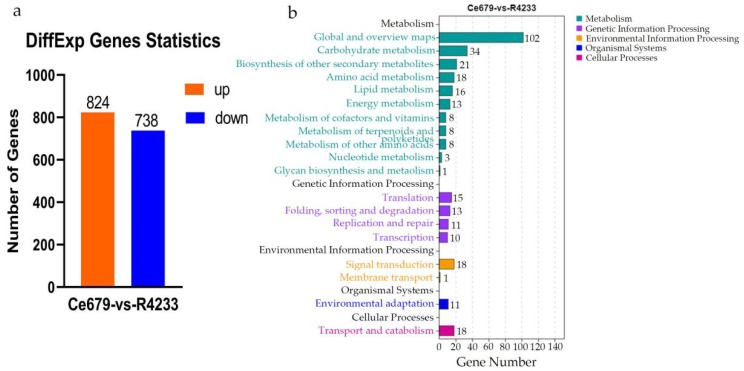
Identification of DEGs and KEGG pathway analysis. (**a**), DEGs in young panicle on Ce679 and R4233. (**b**), KEGG pathway annotation of young panicle on Ce679 and R4233.

The analysis of the three replicated samples collected from young panicles in Ce679 and R4233 allowed the identification of 1562 DEGs, of which 824 and 738 were up and downregulated, respectively, in R4233 vs. Ce679 (Figure 10a). Further, these DEGs were significantly enriched for key metabolism-associated KEGG categories, including “global and overview maps,” “carbohydrate metabolism”, “biosynthesis of other secondary metabolites”, “amino acid metabolism”, “lipid metabolism”, “signal transduction”, and “transport and catabolism” (Figure 10b). Among all DEGs, a total of six galactose metabolism-related genes were selected, including *STS1, GIF1, Os06g0675700*, *OsUGE1*, *RFS2*, and *RS5*. The levels of *RFS2* in R4233 were nine-fold more than those in Ce679, while the *RS5* expression levels in R4233 were thrice more than those in Ce679 (Table S13).

### 3.9. Combined Analysis of DEGs and DEMs

**Figure 11:**
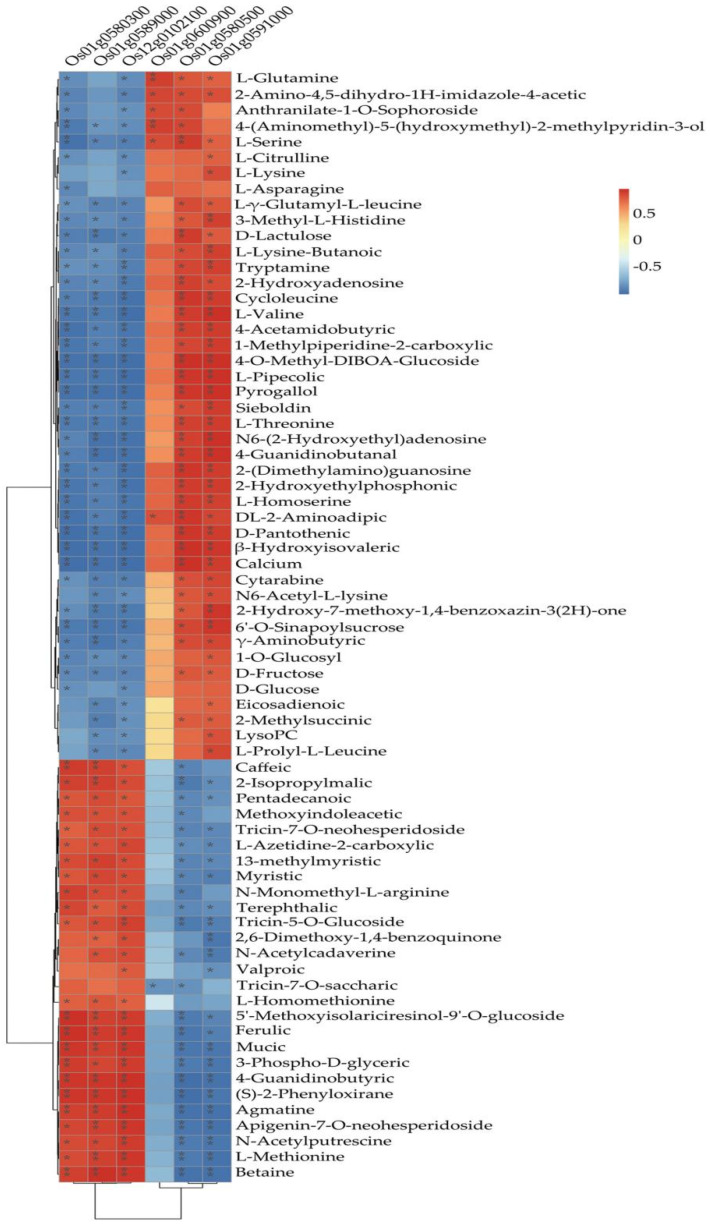
Correlation heat map between 6 DEGs and 71 metabolites; the significant level is indicated with “*”; *, indicates *p* < 0.05; **, indicates *p* < 0.01; unmarked, indicates no significance.

**Figure 12:**
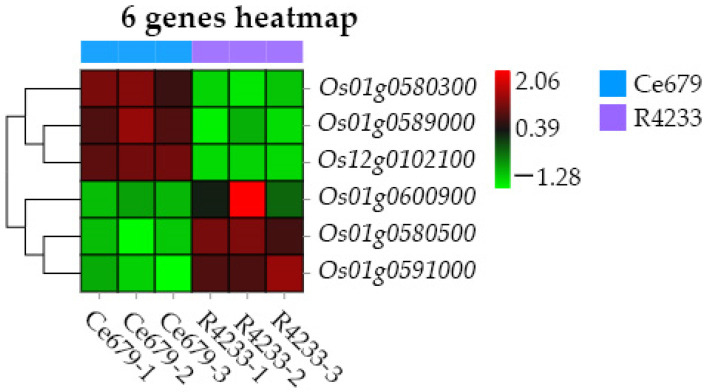
The co-expression network analysis of DEGs and DEMs between Ce679 and R4233 in young panicle.

The heat map showed that the six selected galactose-related genes could be divided into two groups with contrasting metabolites regulation. Group I with three genes (*Os01g0580300*, *Os01g0589000*, *Os12g0102100*), were upregulated in 27 metabolites and down-regulated in 44 metabolites, while group II also with three genes (*Os01g0600900*, *Os01g0580500*, *Os12g0591000*) were upregulated in 44 metabolites and downregulated in 27 metabolites (Figure 11). A subsequent analysis revealed that the genes in group I were downregulated in R4233 in three replicated samples and upregulated in Ce679, while the group II genes were upregulated in R4233 and downregulated in Ce679 (Figure 12).

In our findings, the biosynthesis of amino acids was the most significantly enriched pathway from the metabolite analysis. Moreover, it was revealed that L-Lysine, L-Threonine, L-Homoserine, L-Serine, L-Asparagine, L-Valine, DL-2-Aminoadipic acid, L-Glutamine, and L-Citrulline increased from Ce679 to R4233. In contrast, the levels of 2-Isopropylmalic acid and 3-Phospho-D-glyceric acid were reduced (Figure 9b, Table S12).

**Figure 13:**
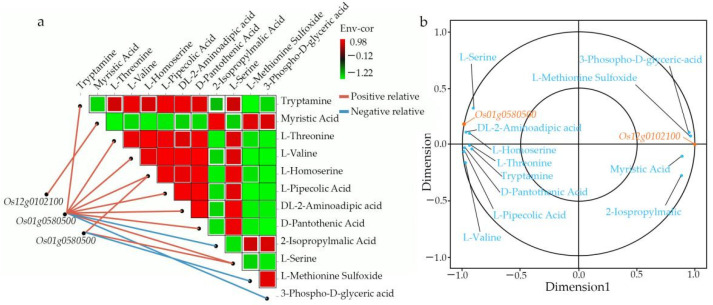
The co-expression analysis of DEGs and DEMs is based on Pearson correlation. Pearson correlation coefficient > 0.8 or ≤ −0.8, *p*-value ≤ 0.05. (**a**), Dynamic network heat map of metabolites and genes; the horizontal and vertical axes represent DEMs, and the red or green color in each square of the heat map indicates the positive or negative correlation coefficient between DEMs; DEGs are shown on the left side, and these genes are correlated with differentially expressed metabolites one by one by connecting lines. The blue and orange lines indicate the degree of significance. (**b**), Canonical correlation analysis (CCA) of metabolites and genes.

We carried out the co-expression network analysis (Pearson correlation coefficient > 0.8 or ≤−0.8, *p*-value ≤ 0.05 (Table S14)) of DEMs and DEGs to highlight the relationship between DEGs and DEMs in young panicles between Ce679 and R4233. The DEGs and DEMs in Ce679 vs. R4233 showed that two genes, *Os12g0102100* and *Os01g0580500*, and 12 metabolites were significantly enriched in three metabolic pathways (biosynthesis of secondary metabolites, cysteine and methionine metabolism, fatty acid biosynthesis). *Os12g0102100* was related to fatty acid biosynthesis and the main product called myristic acid (Figure 13a). *Os01g0580500* coded for the enzyme 1-aminoclopropane-1-carboxylate oxidase (*OsACO7*). Except for fatty acid biosynthesis, the co-expression network of DEGs and DEMs in Ce679 vs. R4233 were mainly enriched in the biosynthesis of primary and secondary metabolites (e.g., L-Serine, L-Valine, L-Threonine, L-Homoserine, L-Pipecolic Acid, Tryptamine, DL-2-Aminoadipic acid, 2-Isopropylmalic Acid, 3-Phospho-D-glyceric acid, D-Pantothenic Acid, *Os01g0580500*) (Figure 13a) and cysteine and methionine metabolism (e.g., L-Serine, L-Homoserine*, L-Methionine Sulfoxide) (Figure 13a). The results showed that the GNPP could be affected by the co-expression of DEGs and DEMs related to fatty acid biosynthesis, biosynthesis of the secondary metabolites, and cysteine and methionine metabolism. The canonical correlation analysis showed that the *Os01g0580500* (*ACO7*) gene had a high correlation with DL-2-Aminoadipic acid and L-Homoserine; the *Os12g0102100* (*At3g45770*) gene had a high correlation with L-Methionine Sulfoxide (Figure 13b).

### 3.10. Validation of Transcriptome Data

**Figure 14:**
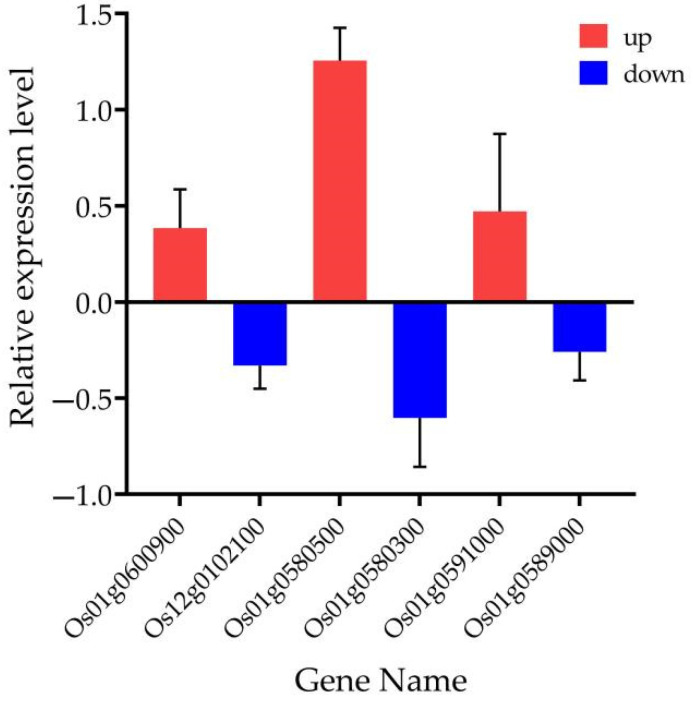
Real-time quantitative PCR validation of transcriptome data for six selected DEGs between Ce679 and R4233. The data were obtained from six independent repeats.

We selected six genes, *Os01g0600900*, *Os12g0102100*, *Os01g0580300*, *Os01g0580500*, *Os01g0591000*, and *Os01g0589000*, to analyze their expression pattern in Ce679 vs. R4233 at the panicle initiation stage to validate the transcriptome experiment results (Figure 14). The RT-qPCR results indicated that the selected genes’ expression pattern was consistent with the RNA-seq data, having similar expression trends despite the quantitative difference in the expression level.

## 4. Discussion

The GNPP is a primary agronomic trait that directly determines rice grain yield. Rice grain constituted one of the principal targets during artificial selection, and improving this trait has been integrated into the selection strategies by breeders and molecular biologists. It is profoundly affected by the panicle architecture-related components, such as the length of the central rachis and the number of primary and secondary rachis branches. The current study investigated two *indica* rice cultivars: Ce679 (low GNPP) and R4233 (high GNPP). R4233 is a restorer line developed from the successive backcrossing of Ce679. After evaluation, the results showed that it has gained some superior agronomic traits from its parents in addition to *Pi9* for disease resistance. The combination of blast disease resistance and grain yield is essential in breeding. Although other traits such as flag leaf length and width that also showed a significant difference in this study are important for photosynthesis, the grain number remains the ultimate trait that can directly impact the food security. Thus, it is reasonable for breeders to seek a cultivar with a better yield.

Since environmentally stable QTLs could be applied in a wide range of circumstances, the segregating population for QTL identification related to the GNPP was developed in the experimental field of Guangxi University and in Bobai Southern China during different growing periods. The quantification of different agronomic traits revealed remarkable differences in the panicle structure in R4233 compared with Ce679. We observed similar results for the GNPP in R4233 regardless of the location, which is important in breeding. The number of secondary branches per panicle was significantly higher in R4233 than the number of primary branches in Ce679 (Table 2), suggesting that the secondary branches significantly contributed to the total branches and grain number. In our results, secondary branches contributed 82.3%, 86.6%, and 87.1% of the total branches in Ce679, R4233, and F1, respectively. The numbers of primary rachis branches and secondary rachis branches between Ce679 and R4233 were significantly different, but no difference was found concerning the length of the central rachis. Therefore, the GNPP in R4233 was significantly higher than Ce679 (Figure 1). Conversely, the number of primary branches per panicle was not significantly different in F1 compared to Ce679. At the same time, this difference was significant in the number of secondary branches per panicle and GNPP. This result indicated that the development of the secondary branch per panicle might be the primary component that affects the GNPP in Ce679 and R4233, causing the grain number to double in R4233 compared with Ce679. In rice, panicle development is critical in grain production; mainly the transition to the reproductive phase, which involves the transformation of the shoot apical meristem (SAM) into the inflorescence meristem (IM). During this period, several lateral meristems (LM) initiate and grow as primary rachis branches (PRBs), which further produce next-order LM that grow as secondary rachis branches (SRBs). Later, the lateral spikelet will differentiate from the new LM, and the terminal spikelets are converted from rachis branch meristems. Several studies reported a positive regulation of the GNPP through the development of only the secondary rachis branch [11,19,46,47], or simultaneously primary and secondary rachis branch-related genes [13,18,48,49]. However, none of the previously identified genes have been found to directly relate to the number of grains in the present study, suggesting another mechanism contributing to the GNPP.

In a recent study, Guo et al. [24] reported that the completion of mapping and cloning of the *Ctb1* gene related to the chilling resistance in rice took over 16 years with conventional breeding and intensive labor [24]. This is because it involved several steps such as fine mapping, map-based cloning, and high-density linkage maps [24]. This procedure has been simplified over the last decades with the development of high-throughput technologies, and the researcher can save much time, labor, and money. Lately, several “omics” techniques, which include, genomics, transcriptomics, proteomics, and metabolomics, have become effective technologies for plant functional genomic and breeding research [50]. Our research took advantage of this technology to accelerate the identification of genes and pathways which take 4 years where more time is needed. Omics research is also undergoing a shift from a single-omics to a large-scale multi-omics approach. In most of the previous research, a single-omics approach was used [51]. For instance, researchers employed BSA-Seq as an effective approach to identify the minor genes with various sequencing depths because of the precision and sensitivity of the sequencing. RNA-Seq was used to identify new genes and SNP loci, measure gene expression levels, and calculate fold changes in DEG. Proteomics was simply to detect gene products and metabolomics measured how proteins are expressed, and the pathway of metabolites, which influence how genes display the biochemical phenotype of the cell. The limitation of the single-omics approach is that it may not help to obtain a deeper understanding of the fundamental biological processes, a more accurate prediction of the response variable, and gain further insight into mechanistic aspects of the system [52]. So, an integration of the different omics approaches is required to envisage overall comprehension of the gene, product, and phenotype under a set of conditions. This approach is gaining more interest and has been successfully used. Recently, in many crops, physiological activity, agronomic traits, responses to biotic and abiotic stress, and yield have been well documented via the use of integrative omic approaches. This robust approach has superseded conventional phenomics, resulting in a formidable tool for crop genetics and breeding sciences [53]. Combining genetic data with prospective phenotyping technologies may offer information on complicated features to help improve crops [54]. The combination of BSA-seq and RNA-seq allowed the identification of the candidate for the agronomic trait [24,28,29,55]. This approach enhanced the accurate identification of gene candidates for the grain number in rice [26], where BSA-Seq alone would identify only the candidate interval. The combination of a transcriptome and metabolome analysis helped to predict molecular mechanisms of genes, and gene networks in crop science. For example, Wang et al. (2019) deciphered the complex response mechanisms involved in heat stress in pepper [56]. The large number of specific response of genes and metabolites highlighted the complex regulatory mechanisms and metabolite networks related to various pathways associated with cold stress after combining a transcriptome and metabolome analysis in tobacco [57] and wheat [54]. Several gene and metabolite networks have been revealed as essential for melatonin-mediated salt tolerance in rice through transcriptome and metabolome investigations [58]. However, the main inconvenience remains the huge datasets generated, which require bioinformatic tools for data mining and organizing [59]. Furthermore, in some case it is necessary to carry out some additional molecular works for functional validation of the candidate gene such as RNAi and Crispr/cas9.

In the current study, we applied a multi-omics approach to investigate the GNPP. BSA-Seq was used to identify QTLs’ position. Further, integration of the BSA-Seq analysis and RNA-sequencing to mine QTL related to spikelet grain number at panicle initiation showed thirty-one DEGs, fourteen of which were located on chromosome 1, four on chromosome 5, eight on chromosome 10, and five on chromosome 12. The result reflected the Manhattan plot analysis from BSA-seq, indicating the success of this joint point approach. A KEGG enrichment analysis of these 31 DEGs and 71 differentially enriched metabolites was performed. Two genes, *Os12g0102100* and *Os01g0580500*, and 12 metabolites were significantly enriched in 3 metabolic pathways. *Os12g0102100*, the alcohol dehydrogenase superfamily zinc-containing protein, is a novel gene, and the contribution to the GNPP is not yet elucidated. This gene was downregulated in the cultivar 4233 and upregulated in Ce679, highlighting its negative effect on the GNPP. The *Os01g0580500* code for the enzyme 1-aminoclopropane-1-carboxylate oxidase (*OsACO7*) is responsible for the final step of the ethylene biosynthesis pathway through the conversion of 1-aminocyclopropane-1-carboxylic acid (ACC) into ethylene in flowering plants under aerobic conditions. This gene was highly expressed in R4233 and downregulated in Ce679, suggesting that this gene positively regulated the GNPP through ethylene synthesis. Increasing evidence indicated that several hormones synthesized by plants, including cytokinin (CK), auxin, abscisic acid (ABA), and ethylene, played a crucial role in developing the panicle and indirectly the GNPP in rice by regulating transcriptional and post-transcriptional activities of GNPP-related genes [6,7,60]. A previous study showed that *OsACO7* was linked to ethylene biosynthesis, enhancing the resistance of young rice plants to the infection of blast fungus [61]. Ethylene is a group of plant growth regulators involved in coordinating numerous plant development processes such as germination, growth, ripening, senescence, and biotic and abiotic stress responses. A recent study showed that it also contributed to important agronomic traits in rice, including the regulation of panicle architecture, grain filling rate, and size [60]. Yin and coworkers (2015), revealed that ethylene deficient mutant mhz5/crtiso had smaller panicles, fewer branches, and more excessive tillers than wild-type plants. In addition, several studies reported that the difference in filling rate between the higher and lower spikelets resulted from the level of ethylene in the two parts. A cultivar with a compact panicle had higher ethylene content than lax-panicle rice [62,63,64]. These suggested that the level of ethylene at the panicle initiation stage may positively affect the development of the primary and secondary branches in R4233.

**Figure 15:**
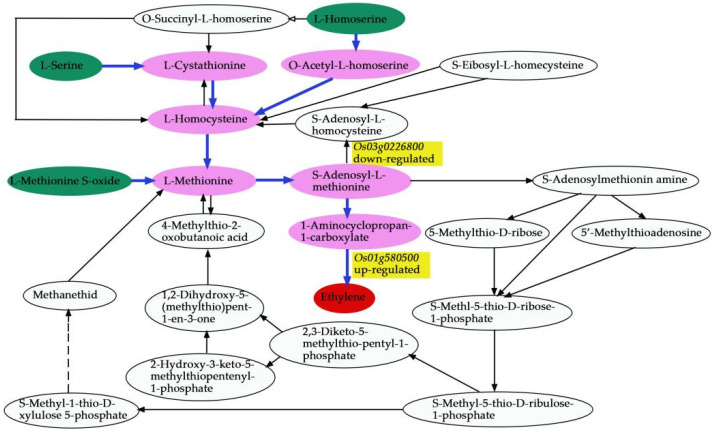
Ethylene formation from L-Serine, L-Homoserine, and L-Methionine Sulfoxide.

We comprehensively assessed the endogenous metabolites involved in these processes. Our results revealed that *OsACO7* interacted with 11 metabolites classified into four groups: amino acids and derivatives (the most prominent group), alkaloids, organic acids, and others sharing two essential pathways. Among the twelve metabolites, ten were found in the biosynthesis of secondary metabolites, suggesting that this pathway contributed substantially to the development of primary and secondary branches of the panicle. This result was in line with Ke et al. (2018), who found that the biosynthesis of secondary metabolites contributed to rice panicle development. Secondary metabolites are generally regarded as indispensable to maintaining normal metabolism and completing the normal life cycle in the plant. Cysteine and methionine metabolism pathways involved three of twelve metabolites: L-Serine, L-Homoserine, and L-Methionine Sulfoxide. Although only three metabolites were identified in this pathway, they seemed to contribute because they can be considered the primary substrate from which ethylene is synthesized (Figure 15). L-Serine and L-Homoserine were also identified in the biosynthesis of the secondary metabolites’ pathway, suggesting that these metabolites were essential in ethylene biosynthesis and the GNPP. Juan and co-authors (2014) reported that ethylene was synthesized from S-adenosylmethionine (SAM) through 1-aminocyclopropane-1-carboxylic acid (ACC), and L-serine was the primary source of one-carbon units for methylation reactions that occurred with the generation of S-adenosylmethionine [65]. Other studies also revealed that ethylene was produced from methionine [66,67].

Another essential pathway was fatty acid biosynthesis, in which the gene *Os12g0102100* coding for mitochondrial trans-2-enoyl-CoA reductase was implicated in the biosynthesis of myristic acid, also called tetradecanoic acid, which is a long-chain saturated fatty acid. Qin and colleagues (2007) showed that saturated, very-long-chain fatty acids promote the development of cotton fiber and the elongation of cells in arabidopsis through the activation of the biosynthesis of ethylene. A previous study reported that myristic acid is vital in cell regulation because it modifies the number of proteins through acylation and N-myristoylation in the signal transduction cascade [68,69]. The high expression of this gene increases the production of miristic acid, which may interfere with the ethylene production pathway in Ce679, leading to the repression of the development process of secondary branches. The differential regulatory mechanism may coincide with the specific ethylene responses to secondary and primary branches’ development, suggesting a possibility for a novel ethylene-GNPP regulatory mechanism in rice.

## 5. Conclusions

The present study coupled different omics approaches to identify potential candidate genes for the GNPP, perform the functional analysis, and investigate the overall pathway in which there is a possible interaction. Our findings revealed that two main QTLs controlled the frequency distribution of the GNPP in the F_2_ population deriving from the crossing between Ce679 (low GNPP) and R4233 (high GNPP) in these cultivars. This result indicates that *Os12g0102100* and *Os01g0580500* might be crucial at panicle initiation, and the GNPP was enhanced through ethylene biosynthesis in rice, which has never been reported. The different phenotypic changes may reflect plant-specific responses to ethylene. Further validation studies, including genetic transformation, RNA interference, and overexpression, may disclose the physiological mechanism which regulates the GNPP in rice. This research showed that a multi-omics analysis is a complementary approach that could help to find important candidate genes for functional investigation. This robust approach is a formidable tool for crop genetics and breeding sciences.

## Figures and Tables

**Table 1:** Comparative analysis of some important agronomic traits between Ce679 and R4233.

Agronomic Traits	Ce679	R4233	Significance
Main panicle length (cm)	25.45 ± 0.75	26.60 ± 0.44	NS
Flag leaf length (cm)	31.20 ± 1.87	42.96 ± 1.49	**
Flag leaf width (cm)	1.91 ± 0.06	2.31 ± 0.03	**
Main stem diameter (mm)	7.97 ± 0.22	8.69 ± 0.28	*
No. of primary branches (per main panicle)	10.60 ± 0.31	15.30 ± 0.42	**
Main stem primary branch length	11.38 ± 0.29	11.97 ± 0.16	NS
Main stem primary branch grain number	16.34 ± 0.75	22.11 ± 0.79	**
No. of secondary branches (per main panicle)	34.40 ± 2.38	69.80 ± 3.43	**
Main stem secondary branch grain number	3.37 ± 0.07	3.57 ± 0.06	NS
Main stem filled grain number	94.90 ± 6.77	147.80 ± 9.72	**
Grain number of the main panicle	173.80 ± 10.72	338.90 ± 16.61	**
Plant height (cm)	99.96 ± 2.08	99.58 ± 1.01	NS
Tiller number	9.20 ± 0.59	8.50 ± 0.37	NS
Grain yield per plant (g)	19.78 ± 0.96	24.32 ± 1.14	**
1000-Grain weight (g)	22.55 ± 0.43	19.26 ± 0.35	*
Number of completely filled grain per plant	92.97 ± 4.54	155.67 ± 9.98	**
Seed setting ratio (%)	78.35 ± 1.93	74.26 ± 1.06	NS
Grain number per panicle	116.94 ± 4.07	224.55 ± 9.87	**
Grain length (mm)	8.88 ± 0.03	8.57 ± 0.04	**
Grain width (mm)	2.59 ± 0.02	2.57 ± 0.02	NS
Length–width ratio	3.49 ± 0.02	3.40 ± 0.01	**

Note: Data in the table were collected from 10 individual plants of Ce679 and R4233 cultivars harvested in the trial conducted in the Nanning experimental field in the late season of 2020. The plant height was measured based on the tallest tiller from the ground to the tip of the panicle. Each trait’s mean value was calculated and compared between Ce679 and R4233. NS means not significant significance level; *, significant at *p* ≤ 0.05 and **, very significant at *p* ≤ 0.01.

**Table 2:** Quantitative evaluation of the structure of the panicle in Ce679, R4233, and F1.

Line	Plant Height (cm)	Flag Leaf Length (cm)	Flag Leaf Width (cm)	Stem Diameter (mm)	Main Panicle Length (cm)	No. of Primary Branches Per Panicle	No. of Secondary Branches Per Panicle	Grain Number Per Panicle
Ce679	120.53 ± 1.51 a	38.04 ± 1.94 b	2.22 ± 0.05 a	5.79 ± 0.20 b	28.07 ± 0.54 a	11.70 ± 0.15 b	54.80 ± 1.93 b	189.00 ± 10.73 b
F_1_	120.64 ± 1.16 a	52.38 ± 1.59 a	2.23 ± 0.07 a	6.69 ± 0.22 a	29.22 ± 0.42 a	12.40 ± 0.42 b	83.50 ± 2.38 a	296.51 ± 11.99 a
R4233	118.28 ± 1.39 a	48.80 ± 1.79 a	2.17 ± 0.03 a	6.35 ± 0.18 ab	29.60 ± 0.52 a	13.70 ± 0.43 a	88.80 ± 1.43 a	313.29 ± 13.77 a

Note: The main panicle was used in the measurement of different traits. Ten plants were harvested from Ce679, R4233, and F1, and the means were calculated with SD (*n* = 10) at 5%. The values with the same letter in the table denote that the difference is not significant, and values with different letters mean the difference is significant (*p* ≤ 0.05).

**Table 3:** Overview of the BSA-seq data.

Sample	Raw Read	Clean Read	Mapped Read (%)	Q30 (%)	GC (%)	Average Depth	Coverage Ratio 1X (%)
Ce679	41,450,665	41,349,765	97.85	94.30	42.02	30	91.43
R4233	49,251,364	49,147,400	97.57	94.29	42.13	35	91.94
L-pool	51,350,279	51,294,200	97.48	92.40	42.72	35	93.70
M-pool	71,424,392	71,355,586	97.77	92.58	42.53	49	94.39
H-pool	48,782,948	48,726,624	97.12	92.27	43.15	33	94.24

**Table 4:** Analysis of candidate regions associated with the GNPP.

Pool	Chromosome Number	Start-Position	End-Position	Size (Mb)	Number of Gene
L-pool vs. H-pool	1	22,285,048	22,696,395	0.41	38
	1	22,832,034	22,893,458	0.06	6
	1	22,931,503	23,684,796	0.75	81
	10	19,687,578	22,594,219	2.91	427
L-pool vs. M-pool	12	20,996	326,632	0.31	44
M-pool vs. H-pool	5	13,886	955,644	0.94	143
Total	-	-	-	5.38	739

**Table 5:** Summary of mapping reads and RNA-Seq.

Sample ID	Raw Reads	Clean Reads (%)	Effective Reads	Total Mapped Reads (%)	Q20 (%)	Q30 (%)	GC (%)
R4233-1	44,629,856	44,429,038 (99.55%)	44,176,010	41,797,107 (94.61%)	97.77%	93.84%	49.09%
R4233-2	42,300,694	42,117,204 (99.57%)	41,957,992	39,469,127 (94.07%)	97.99%	94.33%	48.85%
R4233-3	44,074,652	43,888,866 (99.58%)	43,726,196	41,153,364 (94.12%)	97.96%	94.31%	49.03%
Ce679-1	42,802,616	42,622,640 (99.58%)	42,455,246	40,135,774 (94.54%)	98.00%	94.37%	49.23%
Ce679-2	42,082,242	41,904,188 (99.58%)	41,628,338	39,389,194 (94.62%)	98.04%	94.49%	49.10%
Ce679-3	47,810,372	47,605,970 (99.57%)	47,414,340	44,545,893 (93.95%)	97.94%	94.23%	49.19%

**Table 6:** Expression level of DEGs in the 11 enriched KEGG pathways.

ID	FDR	log_2_ FC	Regulated
*Os01g0580300*	2.04 × 10^−10^	−3.01 × 10^0^	down
*Os01g0580500*	6.49 × 10^−6^	1.18 × 10^0^	up
*Os01g0589000*	7.89 × 10^−10^	−1.12 × 10^0^	down
*Os01g0591000*	3.06 × 10^−10^	1.60 × 10^0^	up
*Os01g0600900*	1.13 × 10^−4^	1.23 × 10^0^	up
*Os12g0102100*	6.55 × 10^−9^	−2.17 × 10^0^	down

**Table 7:** Mapping to reference genome.

Sample	Unmapped (%)	Unique Mapped (%)	Multiple Mapped (%)	Total Mapped (%)
R4233-1	5.39%	91.27%	3.35%	94.61%
R4233-2	5.93%	90.67%	3.40%	94.07%
R4233-3	5.88%	90.69%	3.43%	94.12%
Ce679-1	5.46%	90.98%	3.55%	94.54%
Ce679-2	5.38%	91.02%	3.60%	94.62%
Ce679-3	6.05%	90.44%	3.51%	93.95%

## Data Availability

The raw sequence data reported in this paper have been deposited in the Genome Sequence Archive (Genomics, Proteomics & Bioinformatics 2021) in the National Genomics Data Center (Nucleic Acids Res 2022), China National Center for Bioinformation/Beijing Institute of Genomics, Chinese Academy of Sciences (GSA: CRA007165 and CRA007166) that are publicly accessible at https://ngdc.cncb.ac.cn/gsa; accessed on 13 June 2022. Other data can be provided to researchers on request to the corresponding or first author.

## Supplementary Materials

The following supporting information can be downloaded at: https://www.mdpi.com/article/10.3390/biom12070918/s1. Table S1: Primer sequence for qRT-PCR; Table S2: Construction of bulk pools based on grain number per panicle (GNPP) marked per plant; Table S3: SNP detection and annotation through BSA-Seq analysis; Table S4: Indel detection and annotation of BSA-seq analysis; Table S5: SNPs density in different pools; Table S6: Indels density in different pools; Table S7: The genes annotated in the candidate regions associated with the GNPP identified by the ΔSNP-index method; Table S8: The genes annotated in the candidate regions associated with the GNPP identified by the ΔIndel-index method; Table S9: The genes annotated in the final candidate region associated with the GNPP; Table S10: 738 expressed genes in RNA-seq result from BSA-seq; Table S11: 31 DEGs expression; Table S12: The differential expression metabolites were between Ce679 and R4233; Table S13: Information about DEGs of galactose-related gene; Table S14: The information of co-expression network of DEGs and DEMs.

## Abbreviations

BSA-Seq	Bulked Segregants Analysis Sequencing
RNA-seq	RNA-Sequencing
GNPP	Grain Number Per Panicle
QTL	Quantitative Trait Locus
NGS	Next Generation Sequencing
GWAS	Genome-Wide Association Study
BSA	Bulked Segregants Analysis
DEGs	Differentially Expressed Genes
KEGG	Kyoto Encyclopedia Of Genes And Genomes
DEMs	Differentially Enriched Metabolites
SAM	Shoot Apical Meristem
IM	Inflorescence Meristem
LM	Lateral Meristems
PRBs	Primary Rachis Branches
SRBs	Secondary Rachis Branches
CK	Cytokinin
ACC	1-Aminocyclopropane-1-Carboxylic Acid
GA	Gibberellin Acid
ABA	Abscisic Acid
ET	Ethylene
DEP1	Dense Erect Panicle1
LOG	Lonely Guy
PYL	Pyrabactin Resistance-Like
QQQ	Triple Quadrupole
SNP	Single Nucleotide Polyorphism
InDel	Insertion-Deletion
LC-MS	Liquid Chromatograph-Mass Spectrometer
PCA	Principal Component Analysis
PC1	Principal Component 1
PC2	Principal Component 2
GO	Gene Ontology
RNA	Ribonucleic Acid
DNA	Deoxyribonucleic Acid
FPKM	Fragments Per Kilobase per Millions
QC	Quality Control
VIP	Variable Importance in the Projection
ERB	Early Rice in Bobai
ERN	Early Rice in Nanning
LRN	Late Rice in Nanning
NS	Not Significant
CTAB	Hexadecyl trimethyl ammonium Bromid
